# Measurement of Atmospheric Dimethyl Sulfide with a Distributed Feedback Interband Cascade Laser

**DOI:** 10.3390/s18103216

**Published:** 2018-09-24

**Authors:** Shuanke Wang, Zhenhui Du, Liming Yuan, Yiwen Ma, Xiaoyu Wang, Ruiyan Han, Shuo Meng

**Affiliations:** State Key Laboratory of Precision Measuring Technology and Instruments, Tianjin University, Tianjin 300072, China; wangshuanke@tju.edu.cn (S.W.); yuanliming@tju.edu.cn (L.Y.); yiwenma@tju.edu.cn (Y.M.); wangxiaoyu9453@tju.edu.cn (X.W.); hanruiyan@tju.edu.cn (R.H.); mengsh@tju.edu.cn (S.M.)

**Keywords:** chemical sensor, mid-infrared dimethyl sulfide sensor, tunable laser absorption spectroscopy, distributed feedback interband cascade laser, spectral fitting, multi-species measurement, simultaneous measurement, empirical mode decomposition

## Abstract

This paper presents a mid-infrared dimethyl sulfide (CH_3_SCH_3_, DMS) sensor based on tunable laser absorption spectroscopy with a distributed feedback interband cascade laser to measure DMS in the atmosphere. Different from previous work, in which only DMS was tested and under pure nitrogen conditions, we measured DMS mixed by common air to establish the actual atmospheric measurement environment. Moreover, we used tunable laser absorption spectroscopy with spectral fitting to enable multi-species (i.e., DMS, CH_4_, and H_2_O) measurement simultaneously. Meanwhile, we used empirical mode decomposition and greatly reduced the interference of optical fringes and noise. The sensor performances were evaluated with atmospheric mixture in laboratory conditions. The sensor’s measurement uncertainties of DMS, CH_4_, and H_2_O were as low as 80 ppb, 20 ppb, and 0.01% with an integration time 1 s, respectively. The sensor possessed a very low detection limit of 9.6 ppb with an integration time of 164 s for DMS, corresponding to an absorbance of 7.4 × 10^−6^, which showed a good anti-interference ability and stable performance after optical interference removal. We demonstrated that the sensor can be used for DMS measurement, as well as multi-species atmospheric measurements of DMS, H_2_O, and CH_4_ simultaneously.

## 1. Introduction

Dimethyl sulfide (CH_3_SCH_3_, DMS) is a poisonous and easily explosive volatile organic sulfur compound, which originates not only from numerous production and consumption processes of phytoplankton within the marine eco-system, but also comes from the emissions of volcanoes and vegetation [1,2,3,4]. Moreover, it is a component of the smell produced from cooking certain vegetables, notably maize, cabbage, beetroot and seafood. Thus, DMS exists widely on the ocean’s surface as well as in the atmosphere [5,6,7]. DMS primarily comes from dimethyl sulfoniopropionate, a major secondary metabolite in some marine algae, and oxidized in the marine atmosphere to various sulfur-containing compounds, such as sulfur dioxide, dimethyl sulfoxide, dimethyl sulfone, methanesulfonic acid and sulfuric acid [8,9]. Among these compounds, sulfuric acid has the potential to create new aerosols which act as cloud condensation nuclei; through this interaction with cloud formation, the massive production of atmospheric DMS over the oceans may have a significant impact on the Earth’s climate [10,11,12]. Furthermore, DMS has an odor threshold value that varies from 0.6 to 40 ppb (parts per billion) between different persons and it is highly flammable and irritant to eyes and skin with concentration more than 1 ppm (parts per million) [13,14,15]. In conventional municipal wastewater treatment, incubation of activated sludge has 1–10 mg/L dimethyl sulfoxide produced dimethyl sulfide (DMS) in the headspace gas, a concentrations that exceeded the odor threshold by approximately four orders of magnitude [16]. The concentration of DMS in natural gas field is about 1.8 ppm. Moreover, it is about 6.5 ppm in the exhaust fumes from the chemical plant [17]. Therefore, it is necessary to monitor DMS continuously from ppb to ppm levels for the purpose of environmental protection as well as human safety.

Common methods for detecting DMS include Tunable Laser Absorption Spectroscopy (TLAS) [13], Gas Chromatography (GC) [18], Gas Chromatography-Mass Spectrometry (GC-MS) [19], chemical gas sensors [20,21], Gas Chromatography–Flame Photometric Detection (GC-FPD) [22,23], Fourier Transform Infrared Spectrometer (FTIR) [24], etc. However, whether GC, GC-MS, or GC-FPD, although they have a detection limit down to ppb or ppt (parts per trillion) levels, the DMS should be collected with material resistant to adsorption and oxidation. Furthermore, it requires a complex pre-treatment procedure. These methods are either costly or have a short lifetime. Chemical gas sensors such as ZnO gas sensor are made and tested for concentration as low as 2 ppm of DMS [20]. However, the processing and production steps of ZnO gas sensor are very complicated, and the accuracy of the sensor is affected by ambient humidity. FTIR is used to perform rapid measurement of DMS [24]. However, FTIR is often frustrated by interference from water and carbon dioxide for low spectral resolution. TLAS is a promising technique for trace gas measurement in situ or on line, which is usually used to modulate laser and scan the spectral line; thus, we can get the absorption line of molecules. It can not only realize point sampling measurement with a multi-pass gas cell, but can also be used for remote monitoring with open optical path.

Recently, a Mid-Infrared (MIR) spectral DMS sensor was developed by our group [13]. The sensor boasted a very high sensitivity of 20 ppb with working wavelength of 3367.3 nm located at the *ν*14/*ν*18-band of DMS. However, it was balanced by pure nitrogen and only DMS was detected, rather than a real air condition. Although spectral interference has been considered comprehensively during spectra investigation, practically, the sensitivity of the sensor is deteriorated by the strong spectral interference from H_2_O and CH_4_ when working in practical air condition; there is no other method of data processing for reducing the interference of optical fringes and noise; and the accuracy is affected by internal optical path.

Moreover, based on our previous investigation, the atmospheric DMS concentration is about 28 ppb around a sewage treatment tower and about 500 ppb near an instant noodle factory. Thus, in this paper, to develop a DMS sensor with strong anti-interference capability and stable performances to detect polluted atmosphere in those factories and the surrounding environment, we utilized another candidate spectrum with wavelength of 3336.7 nm located at the *ν*1/*ν*8-band of DMS, and deduced the concentration of DMS, as well as atmospheric interference CH_4_ and H_2_O simultaneously by Multi-Component Spectral Fitting (MCSF). Moreover, we reduced the interference from noise and optical fringes by performing Empirical Mode Decomposition (EMD) and reconstruction to the recorded spectral data. The sensor’s performances were evaluated with common air mixture in laboratory conditions.

The remainder of this paper is arranged in three sections: (Section 2) sensor configuration; (Section 3) sensor performance verification; and (Section 4) conclusions. Each section is divided into several subsections, and all of these present the excellent performance of the sensor.

## 2. Sensor Configuration

We developed a DMS sensor based on wavelength modulation spectroscopy, whose theoretical basis is the Beer-Lambert absorption law [25,26,27,28,29,30,31,32]. When the absorbance *A* < 0.05, according to Beer-Lambert absorption law and optically thin condition, absorption coefficient $\alpha (v)=-\alpha [\bar{\nu }+a\cos(\omega t)]$ is an even periodic function in *ωt* and can be expanded as a Fourier cosine series:

(1) $$-\alpha [\bar{\nu }+a\cos(\omega t)]=\sum_{k=0}^{\infty }H_{k}(\bar{\nu },a)\cos(k\omega t)$$

where $\bar{v}$ is the central frequency of laser; *a* is the modulation amplitude, which is the wavelength modulation of the laser generated by sinusoidal modulation; *ω* is the modulation frequency; *H_k_* are Fourier series; and *k* is the harmonic order. Because the second harmonic signal 2*f* is closely related to the absorption and free of background, typically *k* = 2 is used in Wavelength Modulation Spectroscopy (WMS). Thus, for the mixed second harmonic signal of n types of gases, they can be expressed as follows:

(2) $$H_{2}(\bar{v},a)=-\frac{PL}{\pi }\sum_{i=0}^{n}\chi_{i}\sum_{j=0}^{m}\int_{-\pi }^{\pi }S_{ij}\varphi_{ij}(\bar{v}+a\cos(\theta ))\cos2\theta d\theta$$

where *P* is the pressure, *L* is the length of optical path, *χ_i_* is the mole fraction of the *i*th absorbing species, and *S_ij_* and *φ_ij_* are the line strength and line shape function of the *j*th line of the *i*th absorbing species, respectively. Referring to Equation (2), if the pressure and temperature remain constant, we can ignore the variations of line profile, and it can be expressed as:

(3) $$H_{2}(v,a)=\sum_{i=1}^{n}x_{i}*H_{2_{\_}i_{\_}per}$$

where $H_{2_{\_}i_{\_}per}$ is the second harmonic signal of the *i*th absorbing species in per unit volume concentration, and the magnitude of the absorption-based 2*f* signal, $H_{2}(\bar{v},a)$, which is usually measured by a lock-in amplifier, and described as [23]:

(4) $$S_{2f}\approx \frac{GI_{0}}{2}H_{2}(\bar{v},a)=\sum_{i=0}^{n}\chi_{i}*S_{2f}{}_{\_i\_per}$$

where *S_2f_* is the 2*f* signal that is measured by a lock-in amplifier, *G* is the electro-optical gain of the measurement system, *I_o_* is average intensity of the laser at frequency $\bar{v}$, and *S_2f_i_per_* is the 2*f* signal of *i*th absorbing species in per unit volume concentration. Therefore, *S_2f_* of a mixture theoretically equals the sum of the product of each component’s concentration and its 2*f* signal in per unit volume concentration.

### 2.1. Working Spectral Band Selecting

The MIR fundamental absorption spectra of DMS were investigated in detail in our previous work [13]. By compromise of the absorption intensity, spectral line interference, and convenient laser wavelength, the spectral range located at *ν*14/*ν*18-bandwidth wavelength of 3367.3 nm was preferred, and the strong absorption made the sensor possess much higher measurement sensitivity than other spectral lines.

**Figure 1:**
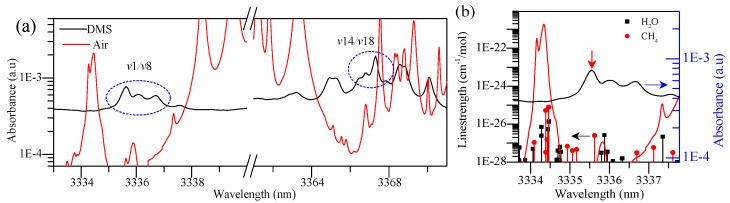
(**a**) Absorption spectra of 5 ppm×m DMS (PNNL database) and air model (H_2_O: 1.860000%, CO_2_: 0.033000%, O_3_: 0.000003%, N_2_O: 0.000032%, CO: 0.000015%, CH_4_: 0.000170%, O_2_: 20.900001%, N_2_: 77.206000%) at 296 K and 1 atm over the range 3333–3371 nm; and (**b**) enlargement of region *ν*1/*ν*8-band of (**a**).

We reexamined the spectral line of DMS based on minimum interference principle, including relatively less interference lines and distant interference spectral lines, and the absorbance of absorption line is relatively large. Thus, another spectral range of DMS was preferred based on the Pacific Northwest National Laboratory (PNNL) and High Resolution Transmission (HITRAN) databases. The 5 ppm×m DMS within the range of 3333–3371 nm is presented in Figure 1a,b.

Figure 1a shows the two regions possible for the measurement of DMS, and Figure 1b is the spectrum located at *ν*1/*ν*8-band to be measured. Detailed analysis is presented in Table 1.

The absorption peak wavelength and strength of DMS candidate regions are listed as well as the spectral information of possible interferences from air absorptions. In Figure 1a,b and Table 1, there are about three main absorption peaks at the *ν*1/*ν*8-band between 3333.7 and 3337.8 nm, and the *ν*1/*ν*8-band of 3336.710 nm suffers much lower interference than *ν*14/*ν*18-band of 3367.229 nm, including the magnitude of interference and distance between the absorption line and the interference line. Thus, we chose 3333.7–3337.8 nm as our ultimate selection for DMS measurement with comprehensive consideration. Furthermore, although CH_4_ and H_2_O have less interference in the absorption of DMS, they are located at the working range of the laser. At the same time, the absorption spectra of methane and water overlap each other, thus it is necessary to obtain the concentration of three gases simultaneously by using the MCSF.

### 2.2. Setup

**Figure 2:**
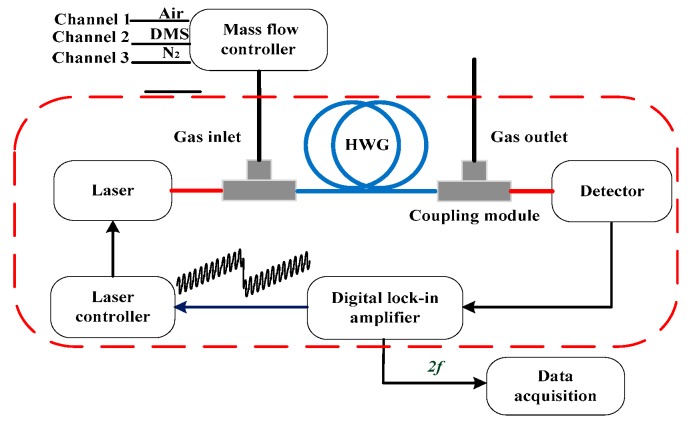
Experimental block diagram.

We developed a DMS gas sensor based on our instrument development platform [25,33,34,35], which consists of a homemade digital lock-in amplifier with signal generation function, a laser controller, a DFB-ICL, a hollow waveguide (HWG), and a photodetector. The schematic plot of the sensor is shown in Figure 2. The digital lock-in amplifier sent out a high frequency modulation signal to the laser controller (ILX Lightwave, Irvine, CA, USA, LDC-3908); the DFB-ICL (Nanoplus GmbH, Gerburnn, Germany) with wavelength of 3337 nm was used as the optical source; and both the temperature and current of the laser were controlled by the laser controller. The beam emitted from the laser was aligned into the HWG (Polymicro Technologies, Brookfield, IL, USA, Type HWEA10001600), and then collected by the photodetector (Thorlabs, Newton, NJ, USA, PDA20H-EC). The converted electrical signal was sent to the homemade digital lock-in amplifier and demodulated. Through data acquisition software, the 2*f* signal was processed by MCSF program to obtain the target gas concentration.

We carefully set all the working parameters of the sensor, including the optical path, gas flow, modulation amplitude, temperature, and so on. The instantaneous line width, dynamic tuning rate, and slope efficiency of ICLs have been reported to be suitable for precision spectroscopy measurements [36]. The laser power before and after HWG were 5.5 mW and 0.9 mW, respectively. Effective optical path length of the HWG was 5 m and its volume was as small as 4.7 mL. The homemade mass flow controller was kept at 50 mL/min with consideration of reducing the influence of pressure changes inside the HWG. The laser controller was controlled by a 10 Hz sawtooth wave with amplitude of 1.45 V and a 2.56 kHz sinusoidal signal with a VPP of 75 mV, and set to 3.5 °C to cover the entire measurement area. At the same time, the digital lock-in amplifier provided a high frequency sinusoidal signal that is two times the frequency of the laser controller to demodulate the spectral signal from the photodetector. Finally, we obtained the 2*f* demodulation signal from the data acquisition software.

We optimized the modulation amplitude of the DFB-ICL to enable measurement of multi-species spectra without overlap. We optimized the modulation amplitude according to Spectral Discrimination (SD) and normalized amplitude of the DMS WMS-2*f* signal [26]. Traditional optimal modulation coefficient (2.2) [29] can no longer meet the requirements because of the interference of noise and optical fringes. Therefore, according to the cross of SD curve and normalized amplitude curve of the DMS WMS-2*f* signal, modulation amplitude of 0.27 cm^−1^ was chose as our optimized selection. Thus, the modulation amplitude of 0.27 cm^−1^ was preferred to improve the Signal-to-Noise Ratio (SNR) and measurement accuracy.

**Figure 3:**
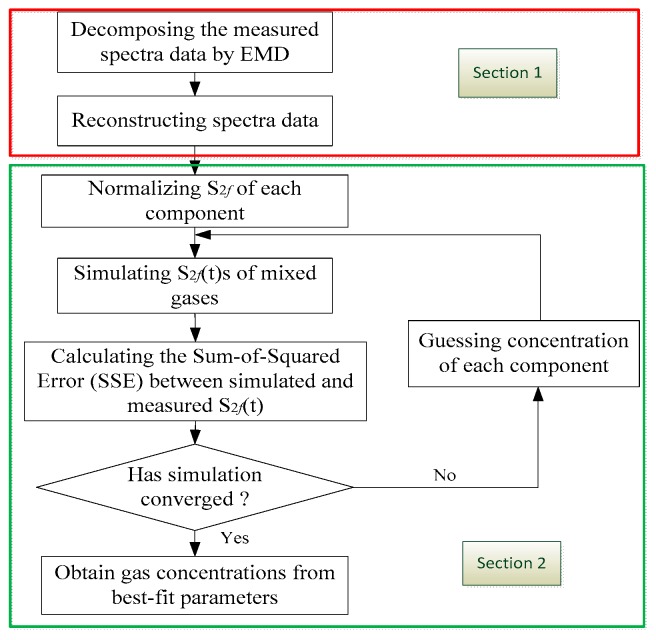
Multi-spectral fitting flow chart [23].

A MCSF algorithm to reduce spectral interference was developed in our previous work [25], as shown in Figure 3. The algorithm is an improved Levenberg-Marquardt (L-M) algorithm, which is based on nonlinear least squares curve-fitting algorithm [28]. In this algorithm, the reference signal of each component and their mixed signals are given first. Because the mixed signals equal the product of the concentration of each gas and their corresponding reference signals, we set the initial concentration of each component and through iterative method, we can get each component’s actual concentration. The algorithm first calculates the concentration of each gas, and then multiplies the corresponding pure absorption signal and sums them. Once the variance between the added mixed signal and the measured signal reaches the minimum, the algorithm converges. After that, the best fitting parameters were the concentrations of all components.

As for the wide-band characteristics of the MIR fingerprint absorption, the measurement sensitivity and accuracy are always deteriorated by overlap with adjacent spectrum, optical fringes, and noise. A solution by EMD and reconstruction to the recorded spectral data was developed in our previous work [26], as shown Section 1 in Figure 3. EMD decomposes the signal into a series of Intrinsic Mode Function (IMF) components; these components have different physical meanings. Thus, we can eliminate optical fringes and noise according to the characteristics of signal, and reconstruct the original signal. In view of the above, we combined the MCSF and EMD to calculate and optimize the multi-component concentration in a mixture. Steps of this algorithm are displayed in Figure 3.

The algorithm includes two parts: (1) EMD is used to decompose and reconstruct the measured spectral data for reducing the optical fringes and noise; and (2) the 2*f* signal of each component is normalized, the initialization concentration parameters of each component are estimated, and the 2*f* signals of single component concentration and mixed concentrations participating in the fitting are produced. If the algorithm converges, the best fitting parameters are obtained. In our simulation, the results of the algorithm basically did not depend on the initial concentration. When the concentration of DMS is within the range of 1–20 ppm, the algorithm always converges.

The initialization concentrations of DMS, methane, and water were set as 1 ppm, 1 ppm, and 1%, respectively. Each component used 370 points to participate in fitting, and the program usually cycles fewer than 100 times. Once the algorithm converges, the best fitting parameters are determined.

### 2.3. Reference WMS-2f Signal Acquisition for MCSF

**Figure 4:**
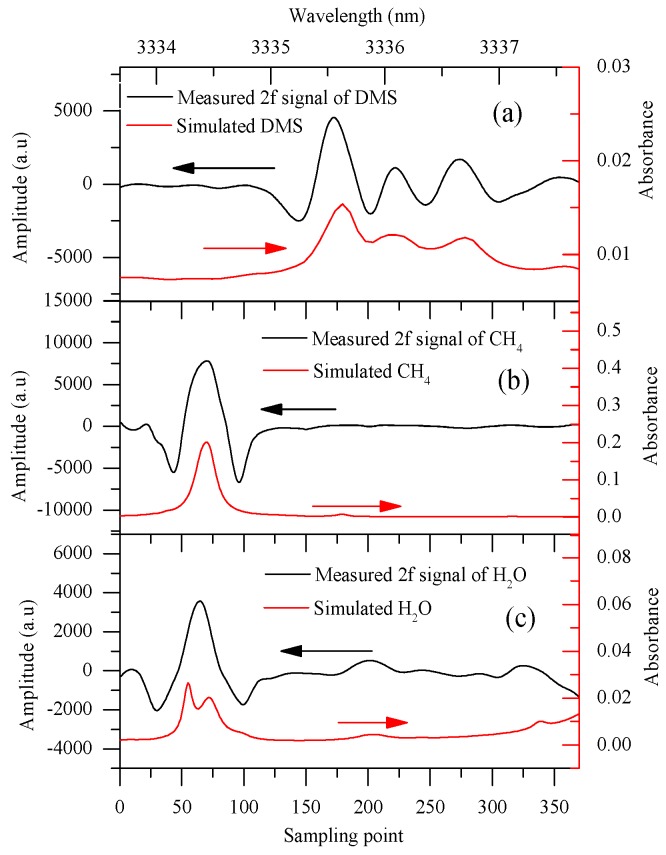
(**a**) The simulated absorbance and reference WMS-2f signals of DMS (20.2 ppm × 5 m); (**b**) the simulated absorbance and reference WMS-2*f* signals of CH_4_ (3 ppm × 5 m); and (**c**) the simulated absorbance and reference WMS-2*f* signals of H_2_O, simulation data come from HITRAN database, 1 atm, 296 K.

We obtained superior reference signals of DMS, CH_4_, and H_2_O for the MCSF algorithm of the sensor with elaborately planned experiments. The HWG was filled with N_2_ first and then with a reference gas, therefore, the difference between the 2*f* signals of the reference gas and N_2_ could be used as reference signal. The reference signals of DMS and CH_4_ were obtained using reference gases with concentrations of 20.2 ppm and 3 ppm, respectively. In contrast, the reference signal of H_2_O was obtained using high humidity air that subtracted air background, and the high humidity air was obtained by a humidifier. With the EMD method [26], we reduced the optical fringes and noise the most. Thus, the measured reference 2*f* signal of each component with less optical fringe and noise between 3333.7 and 3337.8 nm of DMS, CH_4_, and H_2_O are shown in Figure 4a–c, respectively.

### 2.4. Optical Fringes Removal

**Figure 5:**
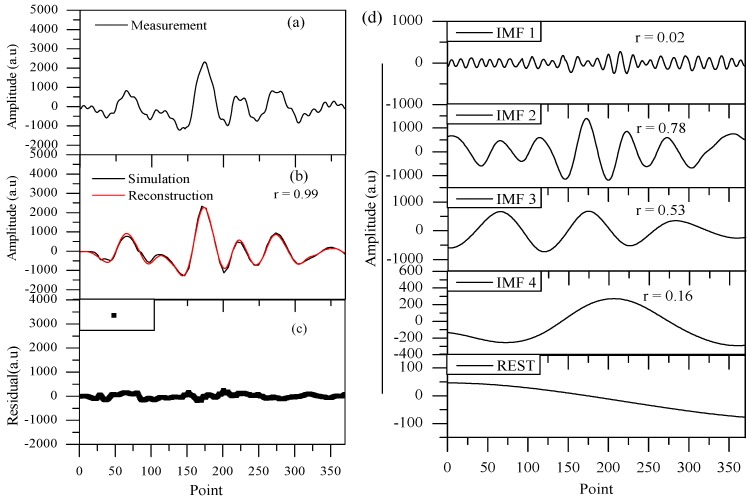
(**a**) The detected 2*f* signals; (**b**) the reconstructed 2*f* signal versus simulated 2*f* signal; (**c**) the residuals between simulation and reconstruction signals; and (**d**) the collection of IMFs that EMD decomposed from the detected 2*f* signal.

We reduced the interference from noise and optical fringes by decomposing the measured 2*f* signals of the mixture and reconstructing them with the EMD method. Take the mixture of H_2_O (0.14%)-CH_4_ (0.19 ppm)-DMS (10.1 ppm) as an example: about four IMF components were obtained. Thus, according to the signal characteristics of correlation coefficient, amplitude, and symmetry, the signal was reconstructed, as shown in Figure 5a–d.

Noise signals usually possess the characteristics of small amplitude and high frequency, while optical fringes are similar to the profile of sinusoidal signal, and all of them have a very low correlation coefficient relative to the absorption signal. Thus, IMF 1 in Figure 5d with a correlation coefficient r of 0.02 was diagnosed as mixture of noise and optical fringes with comprehensive consideration of amplitude, frequency, and correlation coefficient. IMF 2, IMF 3, IMF 4, and the rest of the signal made up the reconstructed absorption signal, as shown in Figure 5b. The reconstructed signal with a correlation coefficient of 0.99 meant that, in the process of decomposition and reconstruction, there was basically no loss or distortion of absorption information.

## 3. Sensor Performance Verification

### 3.1. Detection Ability

**Figure 6:**
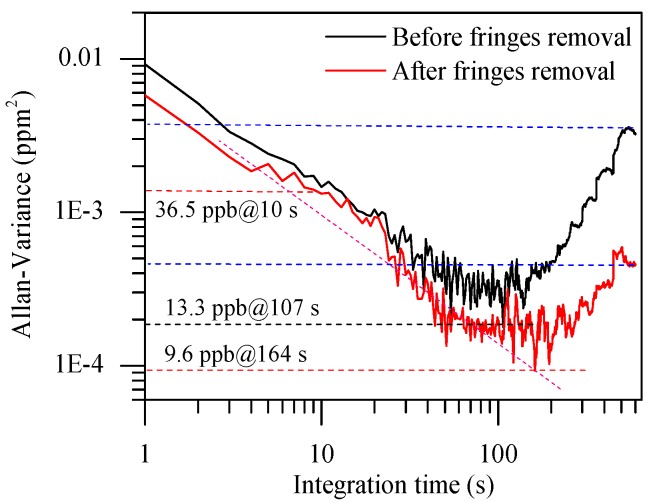
Comparison of Allan variance of the sensor before and after fringes removal.

The detection ability of sensor was usually limited by noise and optical fringes [29,37]. Thus, we tested the detection ability of the sensor with reference gas of DMS before and after fringes removal. We performed EMD, signal reconstruction, and MCSF on recorded spectral signals, and then calculated the concentration of DMS. The calculated concentration was consistent with the nominal value of the reference gas. We evaluated the sensor’s detection ability by calculating the Allan variance of continuous DMS measurements for more than 30 min. To verify the enhancement effect of fringes removal using EMD method, we calculated and compared the Allan variance before and after fringes removal, as shown in Figure 6.

A comparison was made for the sensitivity and detection limit before and after fringe removal. In Figure 6, we can see that the sensor performances have been greatly enhanced, which presented in two aspects: (1) the minimum of the Allan variance reduced from 13.3 ppb (with integral time 107 s) to 9.6 ppb (with integral time 164 s), which means that the sensor’s detection limit significantly improved; and (2) the Allan variance reduced by an order of magnitude when integrated time increased to at least 500 s, as shown in Figure 6 (the two blue dotted lines), which means that the sensor’s long-term stability attained a distinguished improvement. After the removal of fringes, the sensitivity and detection limit of reconstructed signals were optimized at 20 ppb and 3.7 ppb compared to measured signals, respectively. Furthermore, the curve trend of fringes removal indicated that the sensor was much more stable than before, which benefited from the suppression of noise and optical fringes. As seen from the reconstructed signal, there was a detection limit of 9.6 ppb with an optimal integration time of 164 s for DMS, corresponding to an absorbance of 7.4 × 10^−6^, which is sufficient to satisfy the needs of human health. Although the detection limit is much lower than the 2.8 ppb mentioned in Reference [13], it is because of the relatively weak absorption. Actually, our spectral lines performed by EMD have more practical significance, and a greater anti-interference ability, which may also contribute the improvement of detection limit in reference [13].

### 3.2. Measurement Linearity and Uncertainty

Two groups of DMS concentration gradient experiments with air mixture (i.e., CH_4_ and H_2_O), prepared with a homemade high-precision gas mixer [38], were carried out to verify the sensor’s performance. The DMS was prepared with the Gravimetric Standards blending with nitrogen and verified with gas chromatography method [39]. The concentration of CH_4_ and H_2_O in the air were tested beforehand by absolute measurement of direct absorption spectroscopy. The initial concentrations of DMS, CH_4_, and H_2_O were 20.2 ppm, 1.9 ppm, and 1.4%, respectively. Thus: (1) in the first group, the concentration of DMS ranged from 2.02 to 10.1 ppm (in 2.02 ppm intervals), while the concentration of CH_4_ and H_2_O remained at 0.95 ppm and 0.7%, respectively; and (2) the concentration of CH_4_ ranged from 0.19 to 0.95 ppm (in 0.19 ppm), the concentration of H_2_O ranged from 0.14% to 0.7% (in 0.14% intervals), and the volume of DMS remained at 10.1 ppm in the second group. All of the concentration settings were realized by adjusting the three-channel volumetric flow rate settings in Figure 2. Specific reference concentration ratios are shown in Table 2 and Table 3. Each mixture was measured 10 times and the average was taken to reduce noise. The peak absorption of each mixture was estimated to be less than 0.05 and satisfied the optical thin condition of WMS. All experiments were performed at 1 atm.

**Figure 7:**
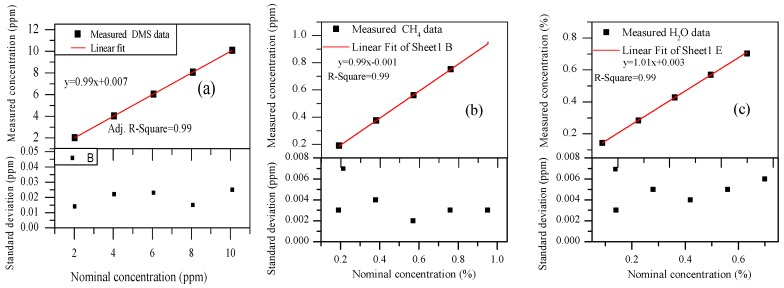
(**a**) The concentration of CH_4_ and H_2_O remained at 0.95 ppm and 0.7%, respectively, while the concentration of DMS ranged from 2.02 ppm to 10.1 ppm (in 2.02 ppm intervals) in the first group, and the standard deviations between nominal concentration and measured concentration are presented; and (**b**,**c**) the volume of DMS remained at 10.1 ppm, the concentration of CH_4_ ranged from 0.19 ppm to 0.95 ppm (in 0.19 ppm intervals) while the concentration of H_2_O ranged from 0.14% to 0.7 % (in 0.14% intervals) in the second group, and the standard deviations between nominal concentration and measured concentration are presented.

We measured and fitted the concentration of DMS in the first group, as shown in Figure 7a, and concentrations of CH_4_ and H_2_O in the second group, as shown in Figure 7b,c, whose concentrations changed in gradient in two groups of experiments. All experiments were carried out at room temperature.

The concentration of DMS, CH_4_, and H_2_O were measured, as shown in Figure 7a–c. The error bars represent the difference between the measured value and the true value. The square of correlation coefficient, R^2^, were all equal to 0.99, which showed a very good linear relationship between the measured concentration and nominal concentration (i.e., reference concentration) for DMS, CH_4_, and H_2_O. The measured accuracy of DMS, CH_4_, and H_2_O were 0.3%, 1.9%, and 2.8%, respectively. The standard deviations could primarily be attributed to the uncertainty of the mass flow controller and the fluctuation of H_2_O in the air over time. Although because of the concentration gradient, the O_2_ and N_2_ concentrations in the experiment are different from their actual concentrations in air, they have no absorption in the spectrum that we chose and have less influence on our measurement.

**Figure 8:**
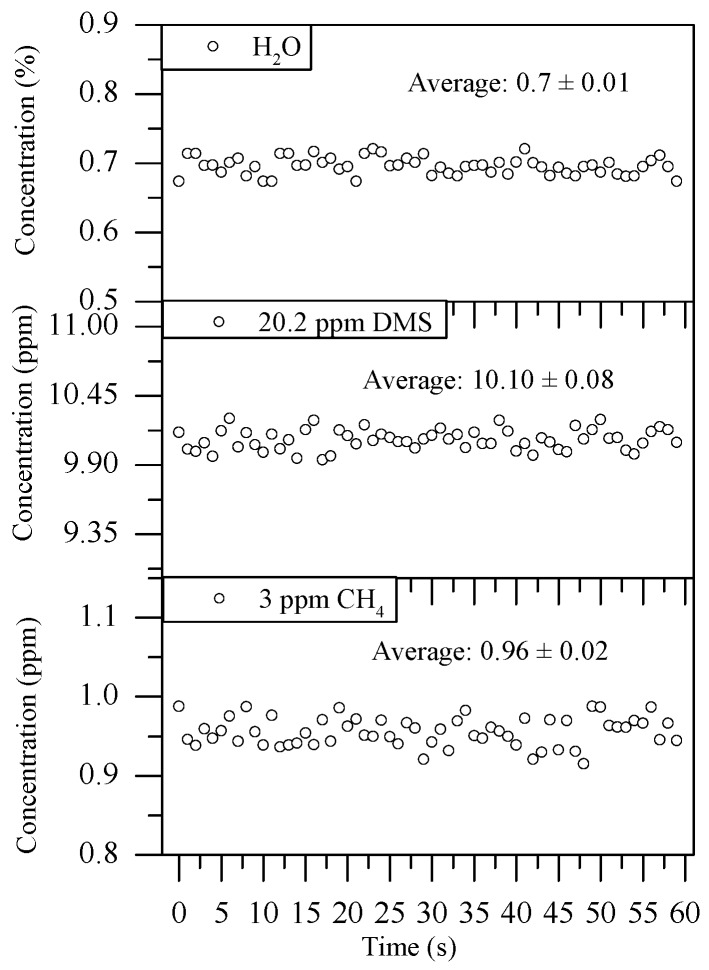
Continuous measurements of DMS, CH_4_, and H_2_O with a duration of 1 min.

Sixty measurements with 1 s intervals were performed to verify the stability and accuracy of the sensor with the flowing gas mixture of 10.1 ppm DMS, 0.95 ppm CH_4_, and 0.7% H_2_O. The results are shown in Figure 8.

In Figure 8, the average concentrations of DMS, CH_4_, and H_2_O were 10.10 ± 0.08 ppm, 0.96 ± 0.02 ppm, and 0.7 ± 0.01% with a sampling interval of 1 s, respectively. Therefore, the measurement uncertainties of DMS, CH_4_, and H_2_O were 80 ppb, 20 ppb, and 0.01%, respectively, which shows that the sensor has an excellent performance.

## 4. Conclusions

In summary, we demonstrated a high-sensitivity and multi-species sensor of atmospheric DMS, CH_4_, and H_2_O. The sensor’s working wavelength located at 3336.7 nm, i.e., the *ν*1/*ν*8-band, was chosen to measure atmospheric DMS, which could avoid serious spectral interference from atmospheric CH_4_ and H_2_O. Multiple techniques were specified for wide-band spectra measurement, including modulation amplitude optimization, MCSF, and removal of optical fringes and noise by EMD, which enhanced the performances of the sensor. Experimental results indicate that the measurement accuracy of DMS was raised to 0.3%, and the measurement uncertainties of DMS, CH_4_, and H_2_O were improved to 80 ppb, 20 ppb, and 0.01%, respectively. Furthermore, detection limit as low as 9.6 ppb with an integration time of 164 s was obtained, thus the sensor can be applied in monitoring the atmosphere around food processing plants and chemical plants. Future work can concentrate on adjusting the structure of optical path and lengthening the HWG, which will further improve the stability and detection limit of the sensor.

## Figures and Tables

**Table 1:** Candidate regions for measurement of DMS concentration based on PNNL and HITRAN databases [13].

DMS Peak Wavelength (nm)	Peak Absorption (1 ppm×m)	λ (nm) (Interference)	Absorbance (Interference)	Distance (Interference)
3336.710	0.118 × 10^−3^	3336.309	0.433 × 10^−5^	0.401
3367.299	0.387 × 10^−3^	3367.554	0.911 × 10^−2^	0.255

**Table 2:** The reference concentration settings of three gases in the first group.

	DMS (Channel 2)		N_2_ (Channel 3)
	Flow (mL/min)	Concentration (ppm)	Flow (mL/min)
Air (25 mL/min, channel 1)	5	2.02	20
	10	4.04	15
	15	6.06	10
	20	8.08	5
	25	10.1	0

**Table 3:** The reference concentration settings of three gases in the second group.

	Air (Channel 1)		N_2_ (Channel 3)
	Flow (mL/min)	Concentration of CH_4_/H_2_O (ppm/%)	Flow (mL/min)
DMS (25 mL/min, channel 2)	5	0.19/0.14	20
	10	0.38/0.28	15
	15	0.57/0.42	10
	20	0.76/0.56	5
	25	0.95/0.70	0
